# Synthetic nickel-containing superoxide dismutase attenuates para-phenylenediamine-induced bladder dysfunction in rats

**DOI:** 10.18632/oncotarget.22395

**Published:** 2017-11-11

**Authors:** Bing-Juin Chiang, Tien-Wen Chen, Shiu-Dong Chung, Way-Zen Lee, Chiang-Ting Chien

**Affiliations:** ^1^ Department of Life Science, College of Science, National Taiwan Normal University, Taipei City 11677, Taiwan; ^2^ Department of Urology, Cardinal Tien Hospital, New Taipei City 23148, Taiwan; ^3^ Department of Urology, Far-Eastern Memorial Hospital, New Taipei City 220, Taiwan; ^4^ Graduate Program in Biomedical Informatics, College of Informatics, Yuan-Ze University, Chungli 320, Taiwan; ^5^ Department of Chemistry, College of Science, National Taiwan Normal University, Taipei City 11677, Taiwan

**Keywords:** urinary bladder, para-phenylenediamine, Ni-SOD mimics, micturition, programmed cell death

## Abstract

Para (p)-phenylenediamine and its toxic metabolites induce excess reactive oxygen species formation that results in bladder voiding dysfunction. We determined the effects of synthetic Ni-containing superoxide dismutase mimics and the role of oxidative stress in p-phenylenediamine-induced urinary bladder dysfunction. P-phenylenediamine (60 μg/kg/day) was intraperitoneally administered for 4 weeks to induce bladder injury in female Wistar rats. Synthetic Ni-containing superoxide dismutase mimics, WCT003 (1.5 mg/kg) and WCT006 (1.5 mg/kg), were then intraperitoneally administered for 2 weeks. Transcystometrograms were performed in urethane-anesthetized rats. The *in vitro* and *in vivo* reactive oxygen species levels and pathological changes in formalin-fixed bladder sections were evaluated. Western blotting and immunohistochemistry elucidated the pathophysiological mechanisms of oxidative stress-induced apoptosis, autophagy, and pyroptosis. P-phenylenediamine increased voiding frequency, blood and urinary bladder levels of reactive oxygen species, and neutrophil and mast cell infiltration. It also upregulated biomarkers of autophagy (LC3 II), apoptosis (poly (ADP-ribose) polymerase), and pyroptosis (Caspase 1). WCT003 and WCT006 ameliorated reactive oxygen species production, inflammation, apoptosis, autophagy, pyroptosis, and bladder hyperactivity. P-phenylenediamine increased oxidative stress, inflammatory leukocytosis, autophagy, apoptosis, and pyroptosis formation within the urinary bladder. Novel synthetic nickel-containing superoxide dismutase mimics relieved p-phenylenediamine-induced bladder inflammation and voiding dysfunction.

## INTRODUCTION

Para-phenylenediamine (PPD) is a common ingredient in hair and leather dyes [1, 2]. Approximately 33% of women over age 18 and 10% of men over age 40 in North America and Europe use hair dye [3]. PPD poisonings have been reported in developing countries due to its widespread industrial application [4, 5]. PPD is defined as an allergen by the American Centers for Disease Control and Prevention that can induce angioneurotic edema, renal, hepatic, and cardiac injury [5], and type IV delayed hypersensitivity [6]. The International Agency for Research on Cancer (IARC) suggested that there was inadequate evidence that personal use of hair dye entails carcinogen exposure despite higher incidence bladder cancer, non-Hodgkin's lymphoma, multiple myeloma, and hematopoietic cancers of hair dye users and colorists [2, 7–9]. People receiving tattoos could bear a higher risk of PPD absorption [10]. The significant public health impact of widespread applications of PPD and the associated health risks should be considered.

PPD is metabolized by cytochrome P450 through peroxidase electron oxidation to an active radical and forms reactive benzoquinone diamine. It is further oxidized to Brandowaski's base, which is highly toxic, mutagenic, and an allergen [5]. PPD can induce production of excess oxygen species (ROS) that impair keratinocytes, germ cells, urothelial cells, and kidney cells through oxidative stress [6, 11–14]. Oral PPD induced acute kidney injury, including glomerular and tubulointerstitial pathological changes, at a sub-lethal (40 mg/kg once) or minimal (20 mg/kg once) dose in a rat model [15]. PPD can also increase expression of oncoprotein p53, cyclooxygenase 2, and autophagy stimulation in human uroepithelial cells [16, 17]. PPD induced apoptosis by activating the ROS-mediated mitochondrial pathway in human urothelial cells [14] and stimulated secretion of vascular endothelial growth factor in keratinocytes to increase microvascular hyperpermeability via a hypoxic mechanism [18]. ROS formation could also upregulate several biomarkers of autophagy (Beclin-1 and LC3 II), apoptosis [Caspase 3 and poly (ADP-ribose) polymerase (PARP)], and pyroptosis (Caspase 1 and IL-18) in chronic ischemic cells and tissues [19] and contribute to bladder inflammation, injury, and hyperactivity [20]. We hypothesized that PPD toxicity could induce hypoxic/ischemic conditions, excess ROS formation, inflammation, and cell death in damaged bladders.

Antioxidants, such as vitamin C, are effective for mitigating PPD-induced allergic contact dermatitis [21]. Superoxide dismutases (SODs) are the main antioxidative enzymes that directly scavenge potential harmful oxidizing species. SODs are categorized by the metal center: manganese (Mn-SOD), iron (Fe-SOD), copper/zinc (Cu/Zn-SOD), and nickel (Ni-SOD). A valine-to-alanine substitution at amino acid −9 in the Mn-SOD gene modified the risk of PPD-induced contact dermatitis in a clinical trial of older females [22], which suggested a potential role for SODs to mitigate PPD toxicity. A novel Ni-SOD recently isolated from *Streptomyces* and marine cyanobacteria catalyzes the dismutation of O_2_^−^ into O_2_ and H_2_O_2_ through a Ni^2+^/Ni^3+^ oxidation cycle [23]. Synthetic Ni^2+^ compounds are known to be more stable than Mn^2+^ compounds, and several synthetic Ni compounds have been reported [24, 25]. However, none have demonstrated the biomedical application of Ni-SOD. We evaluated two modified synthetic mimics of Ni-SOD, WCT003, and WCT006, which possessed similar enzyme activity and may attenuate the inflammatory response from PPD-induced bladder dysfunction in a rat model.

We designed an animal model to evaluate ROS production and bladder function through the chronic administration of minimal doses of PPD. We determined the underlying mechanisms and used novel synthetic Ni-SOD mimics (WCT003 and WCT006) to treat PPD-induced bladder dysfunction.

## RESULTS

### Ni-SOD mimics ameliorated PPD-induced bladder hyperactivity

**Figure 1:**
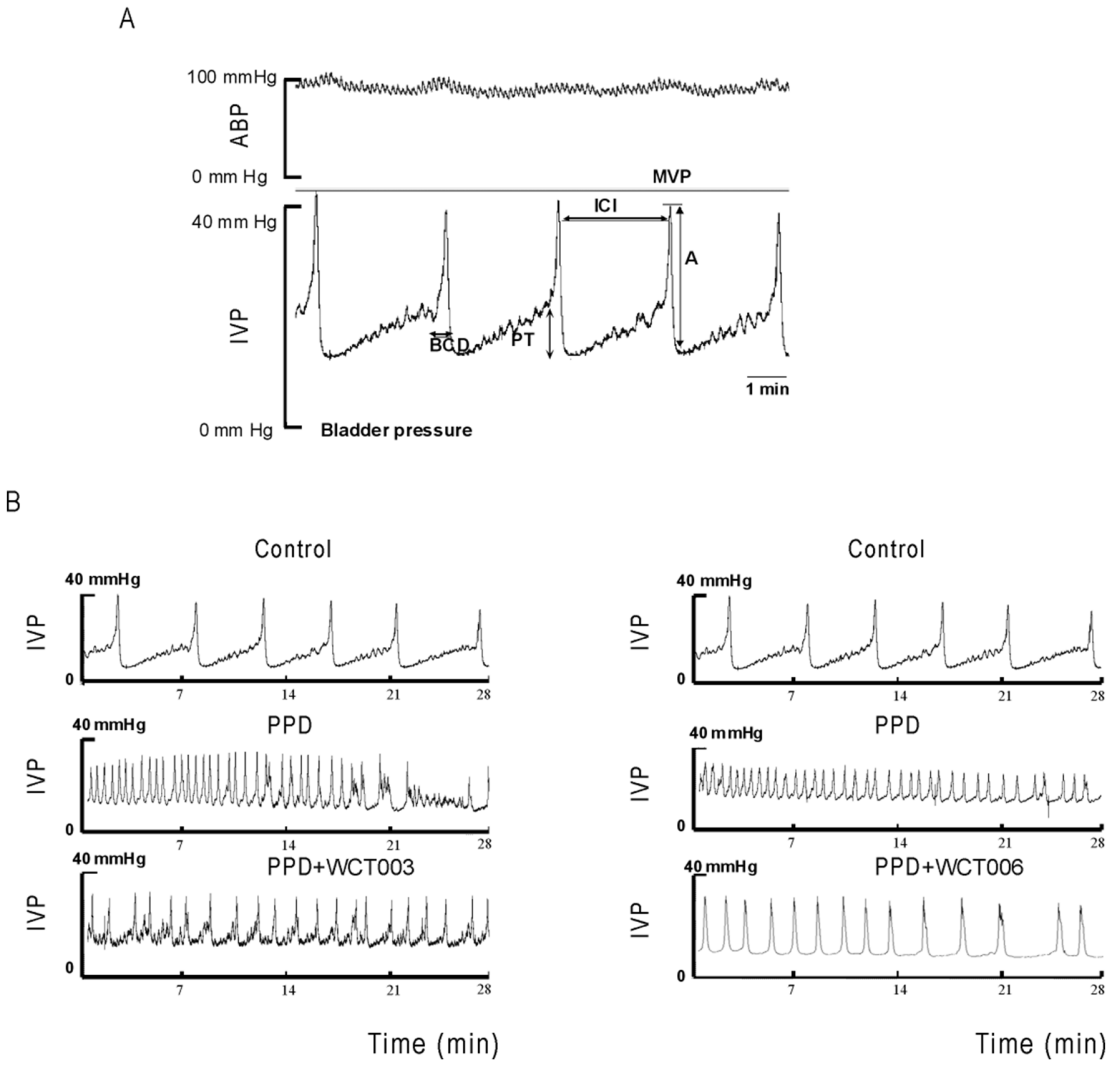
Effect of 4-week PPD treatment on micturition parameters **(A)** The demonstration of voiding parameters, **(B)** PPD treatment induced an overactive bladder and increased the voiding frequency. ABP, arterial blood pressure; IVP, intravesical pressure; MVP, maximal voiding pressure; BCD, bladder contraction duration; A, amplitude; PT, pressure threshold; ICI, intercontraction interval.

**Figure 2:**
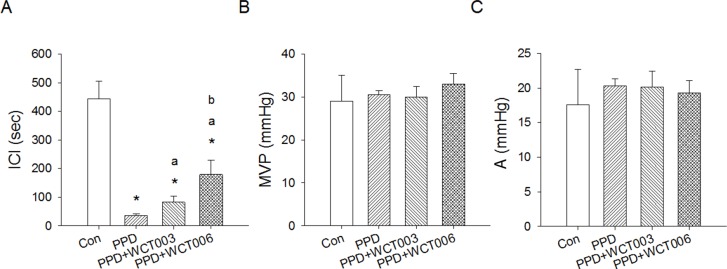
Effect of Ni-SOD mimics, WCT003 and WCT006, on urodynamic parameters of PPD-induced bladder hyperactivity **(A)** PPD treatment for 2 weeks at a dose of 1.5 mg/kg induced bladder hyperactivity with a significant reduction in intercontraction interval (ICI). WCT003 or WCT006 significantly improved the PPD-reduced ICI levels compared to the PPD group. WCT006 increased ICI levels more than WCT003. There was no difference in MVP **(B)** or A **(C)** among the groups of Control, PPD, PPD+WCT003, and PPD+WCT006 rats (n=6 each). A, amplitude=MVP-BP; BP, baseline bladder pressure; ICI, intercontraction interval; MVP, maximal voiding pressure. ^*^P < 0.05 vs. Control group. ^a^*P* < 0.05 vs. PPD group. ^b^*P* < 0.05 vs. PPD+WCT003 group.

Table 1 lists the metabolic cage study results. Rats in the PPD group experienced a higher voiding frequency and greater water intake, urine, and stool amounts than those in the control group (*P* < 0.05). Figure 1 shows the cystometric patterns between the control and PPD groups. PPD treatment induced an overactive bladder with increased voiding frequency. Table 2 and Figure 1 reveal the effects of Ni-SOD mimics on the urodynamic parameters of PPD-induced bladder hyperactivity. The results showed significantly shortened ICI in PPD group (*P* < 0.05). After WCT003 or WCT006 treatment (Figure 2), the ICI in both groups significantly increased compared to PPD group (*P* < 0.05). The MVP and amplitude were not significantly different between the four groups.

**Table 1:** Comparison of baseline parameters between the control and PPD group in metabolic cage studies

	Control group	PPD group
Urinary frequency (24 hours)	33.9±4.2	53.1±13.3^*^
Body weight (gm)	235.7±16.2	277.8±3.8
Water intake (mL)	47.9±8.3	57.7±7.2^*^
Food (gm)	27.1±2.8	22.9±4.4
Urine (mL)	25.6±5.1	32.7±7.5^*^
Stool (gm)	24.3±2.6	35.7±8.1^*^

^*^P < 0.05.

**Table 2:** Comparison of cystometric parameters of four groups

	Control group	PPD group	PPD+WCT003 group	PPD+WCT006 group
ICI (second)	444.0±62.8	35.8±6.0^*^	82.3±13.6^*^	179.0±50.6^*^
MVP (mmHg)	28.5±6.5	30.6±0.9	29.9±2.6	33.1±2.4
A (mmHg)	17.7±5.1	20.3±0.9	20.1±2.3	19.3±1.8

ICI, intercontraction interval; MVP, maximal intravesical pressure; A, amplitude.

^*^P < 0.05.

### PPD-induced bladder ischemia, inflammatory response, and ROS generation in blood

**Figure 3:**
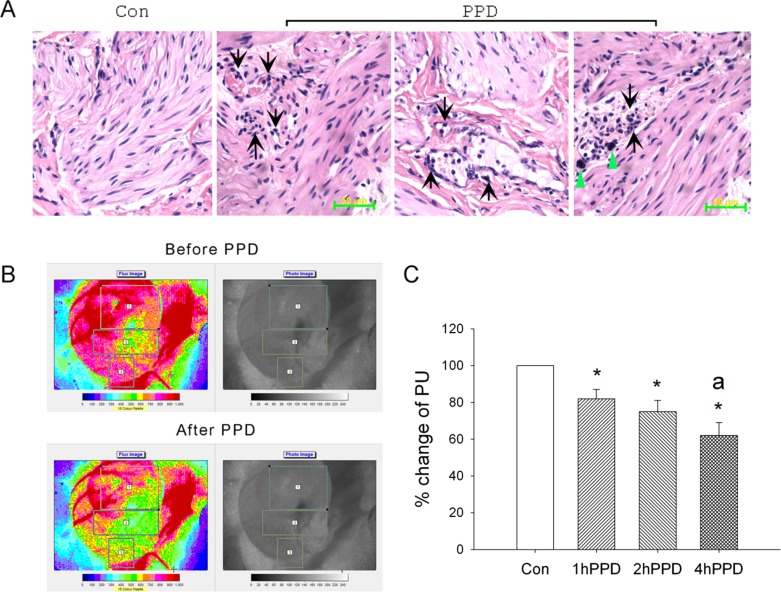
Effect of acute PPD exposure on bladder pathology and microcirculation in the rats **(A)** PPD increased neutrophils infiltration and mast cell appearance in the bladder. There was low infiltration of neutrophils and mast cells in the control (Con) bladder. **(B)** Typical laser-speckle imaging perfusions are displayed on a 16-level color palette in one rat receiving saline (before PPD) and PPD treatment (after 4 h of PPD treatment). **(C)** The mean changes of perfusion unit (PU) are indicated (n=6). ^*^P < 0.05 vs. the data of control status with saline treatment. ^a^*P* < 0.05 vs. 1hPPD. 1 h PPD, after 1 h of PPD treatment; 2hPPD, after 2 h of PPD treatment; 4hPPD, after 4 h of PPD treatment.

Pathological examinations showed that PPD significantly increased neutrophil infiltration and mast cell appearance in the bladder (Figure 3A). Typical laser-speckle imaging perfusions are displayed on a 16-level color palette in one rat receiving saline (before PPD) and PPD treatment (after 4 h of PPD treatment), respectively, in Figure 3B. The mean change in perfusion between control and PPD groups are shown in Figure 3C. The results demonstrated that PPD treatment caused significant bladder ischemia.

**Figure 4:**
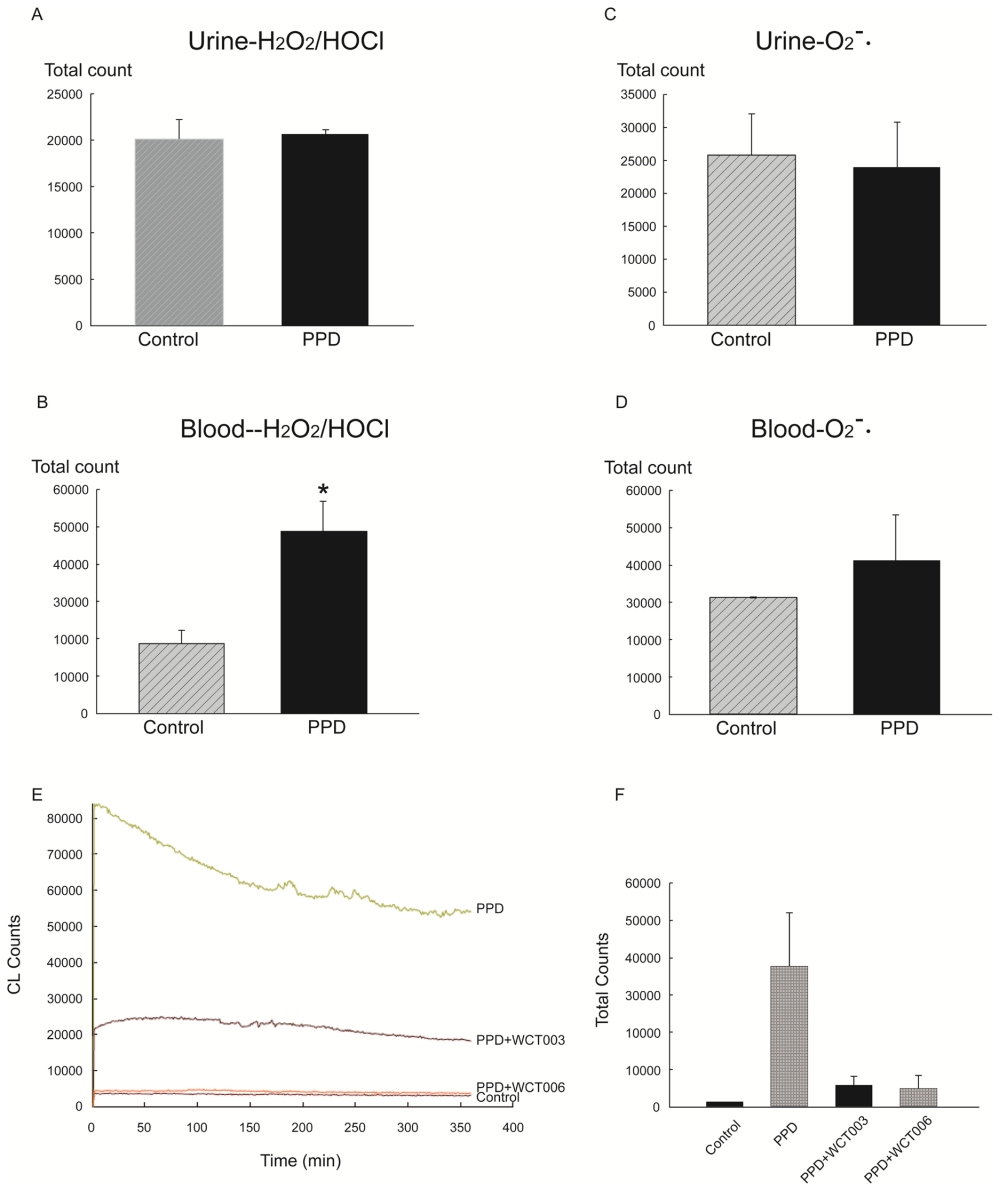
*In vitro* effect of PPD on H2O2 and HOCl activity in urine and blood **(A)** Urine H_2_O_2_ count was not significantly different between the PPD and control group. **(B)** Urine HOCl count was not significantly different between the PPD and control group. **(C)** Blood H_2_O_2_ count was significantly increased in the PPD group compared to the control group (*P* < 0.05). **(D)** There is a trend of high HOCl counts in the PPD group as compared to control group without significant differences. (*P*>0.05). **(E)** The original data of *in vivo* MCLA ROS from the urinary bladder in the control, PPD, PPD+WCT003, and PPD+WCT006 groups. Higher chemiluminescence counts were found in the PPD group compared that in the control group. WCT003 and WCT006 reduced the ROS chemiluminescence counts. **(F)** The statistical data of *in vivo* ROS from the urinary bladder of the control, PPD, PPD+WCT003, and PPD+WCT006 groups. The *in vivo* ROS chemiluminescence count was significantly higher in PPD group compared to that of the control group. (^*^P < 0.05). The *in vivo* ROS chemiluminescence counts were significantly lower in the PPD+WCT003 and PPD+WCT006 groups compared to that of the PPD group. (^a^*P* < 0.05)

The chemiluminescence counts were determined by the area under the curve given by the chemiluminescence detector. *in vitro* urinary ROS generation, indicated by luminol- or lucigenin-amplification methods for measurement of H_2_O_2_/HOCl or O_2_¯, respectively, was not significantly different between control and PPD groups (Figure 4A and 4B). However, PPD significantly increased the blood H_2_O_2_/HOCl count in the PPD group compared that of the control group (Figure 4C). A trend of high O_2_¯ counts in the PPD groups was noted when compared to that of the control group, but the difference was not significant (Figure 4D).

The continuous intravenous infusion of MCLA into a control bladder resulted in a basal level of *in vivo* ROS chemiluminescence at approximately 2500–4000 counts/10 s. In PPD bladders, elevated MCLA-enhanced chemiluminescence was observed and maintained at 55000–85000 counts/10 s (Figure 4E). Treatment with WCT003 or WCT006 significantly depressed the O_2_¯ counts *in vivo* when compared to that of the control group (Figure 4E and 4F). WCT006 was more efficient than WCT003 in reducing the PPD-enhanced O_2_¯ counts.

### Ni-SOD mimics attenuated PPD-induced bladder inflammation, oxidative injury, apoptosis, autophagy, and pyroptosis

**Figure 5:**
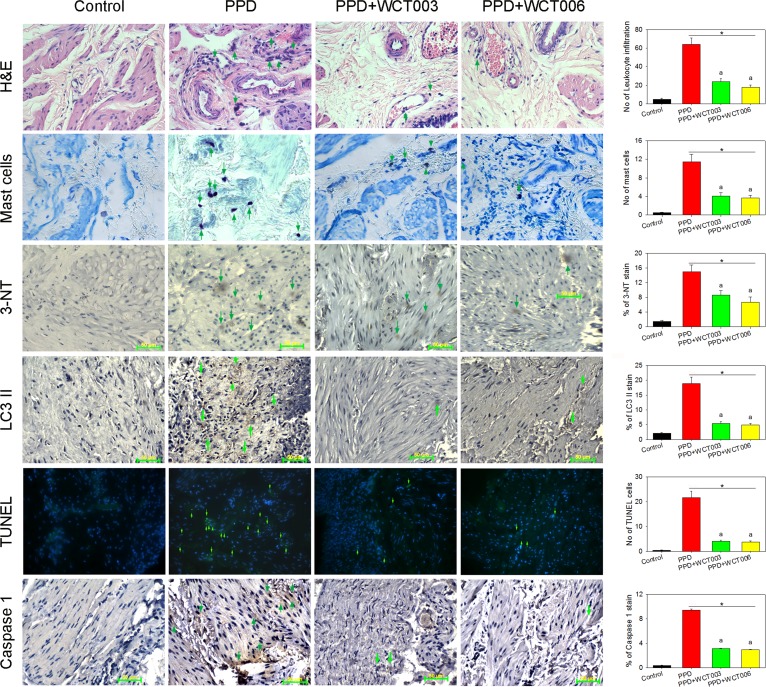
Effect of WCT003 and WCT006 treatment on PPD-induced bladder oxidative injury PPD-enhanced histological changes by haematoxylin and eosin stain, mast cell appearance, 3-NT-mediated oxidative stress as well as LC3 II, TUNEL-apoptosis, and caspase 1 staining (all parameters indicated by green arrows) in the damaged bladders compared to the respective control sections. Intraperitoneal WCT003 or WCT006 effectively reduced PPD-induced pathological parameters. The statistical data are indicated in the right panels. ^*^P < 0.05 compared to control group. ^a^*P* < 0.05 WCT003 or WCT006 vs. PPD group. The scale bar is 50 μm.

**Figure 6:**
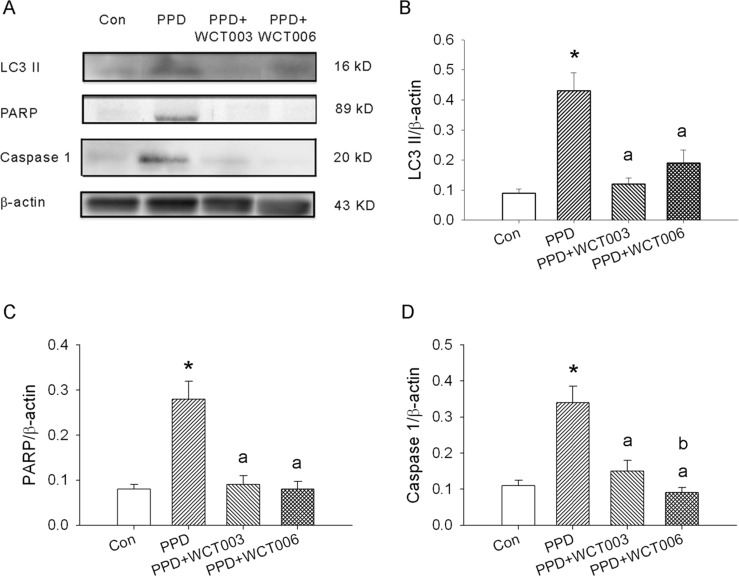
Effect of WCT003 and WCT006 treatment on PPD-induced LC3 II, PARP, and caspase 1 expression Ni-SOD mimics significantly attenuated PPD-induced LC3 II, PARP and caspase 1 expression indicated by western blotting. **(A)** β-actin displayed equal protein loading. The expression of LC3 II, PARP, and caspase 1 were enhanced in the PPD group. WCT003 or WCT006 attenuated the PPD-enhanced expression of LC3 II, PARP and caspase 1. **(B)** LC3 II expression was significantly higher in the PPD group compared to the control group (^*^P < 0.05). PPD-enhanced LC3 II expression in the PPD+WCT003 or PPD+WCT006 groups was significantly lower than the PPD group (^a^*P* < 0.05). **(C)** PARP expression was significantly higher in the PPD group compared to the control group (^*^P < 0.05). PPD-enhanced PARP expression in the PPD+WCT003 or PPD+WCT006 groups was significantly lower than the PPD group (^a^*P* < 0.05). **(D)** Caspase 1 expression was significantly higher in the PPD group compared to the control group (^*^P < 0.05). PPD-enhanced caspase 1 expression in the PPD+WCT003 or PPD+WCT006 groups was significantly lower than the PPD group (^a^*P* < 0.05). Caspase 1 expression in the PPD+WCT006 group was significantly lower than that in the PPD+WCT003 group (^b^*P* < 0.05).

We hypothesized that PPD could induce inflammation, oxidative stress, and three types of programmed cell death (apoptosis, autophagy, and pyroptosis) in damaged bladders. As shown in the left panel of Figure 5, statistical data from the bladder sections revealed that PPD increased the leukocyte infiltration and mast cell numbers as well as oxidative stress, autophagy, apoptosis, and pyroptosis indicated by staining assays in the damaged bladders. Treatment with WCT003 or WCT006 decreased inflammation, oxidative stress markers, apoptosis, autophagy, and pyroptosis in the PPD bladders (Figure 5). We determined the marker expressions of autophagy (LC3 II), apoptosis (PARP), and pyroptosis (caspase 1) in the bladders by western blot. Figure 6 reveals the original and quantitative western blot of apoptosis-, pyroptosis-, and autophagy-related protein expression. PPD significantly enhanced the ratios of LC3II/β-actin, PARP/β-actin, and caspase 1/β-actin in the bladders. Conversely, WCT003 or WCT006 effectively depressed the ratios of LC3II/β-actin, PARP/β-actin, and caspase 1/β-actin in the PPD-damaged bladders. Notably, PARP expression is localized in the cell nucleus, but its expression can be determined in the whole tissue homogenates [26].

## DISCUSSION

PPD penetrates the skin and can be detected in plasma and urine after topical application of a PPD-containing hair dye [27]. The pharmacokinetics and metabolism following dermal application of radioactively labeled PPD were previously investigated. Systemic exposure to PPD varies according to the amount of topical application and duration of exposure [27–29]. The level of systemic PPD exposure for an individual hair dye user is low and is less than 1% of the topical application [29]. There is inadequate evidence that personal use of hair dye entails carcinogenic exposure [7], and a recent comprehensive meta-analysis of epidemiologic studies did not indicate a causal association between personal hair dye use and bladder cancer [30]. However, occupational exposure as an industrial worker and tattoo consumer is more difficult to estimate. The PPD concentration in a black henna tattoo mixture was higher than that of commercial hair dye preparations [31]. Animal experiments revealed that approximately one-third of colorants disappeared from the skin within weeks after tattooing [32]. Furthermore, acute and chronic PPD poisoning in humans has caused renal, hepatic, and cardiac complications [15, 33]. Further assessment of the health risk of PPD is imperative due to its widespread application, despite the low risk from the use of commercialized PPD-contained products.

The highest PPD-derived radioactivity was present in the muscle, skin, and liver of rodents after exposure, while clearance of PPD-derived radioactivity was primarily through the urine (68-86%) and secondarily through the feces (10-19%) [34]. Previous studies demonstrated that PPD led to acute renal failure [15] and impaired urothelial cells through ROS-mediated apoptosis and autophagy formation [17–19]. Our data suggested that toxic metabolites from PPD might impair renal tubular cells by inhibiting water absorption, resulting in early stage diuresis and late-stage oliguria with damaged urothelial cells leading to frequent urination by undefined mechanisms. Increased water intake may be a compensation response for the increased urine output in our rat model.

The previously determined lethal oral dose of PPD for rats was 80 mg/kg body weight [35] and lethal intraperitoneal dose was 37 mg/kg body weight [36]. The highest cumulative penetration of PPD obtained was 4.47 g/cm^2^, which yielded a 0.052 mg/kg systemic exposure dose (close to 60 μg/kg) [35]. Intermittent exposure to low-concentration PPD could be equivalent to a single, higher-dose exposure [37]. Acute PPD intoxication by oxidative stress, a direct toxic effect, and lipid peroxidation-induced bladder injury [14, 16], kidney injury [13, 15], hematological alteration [38], and testicular injury [11, 39] have been observed following sub-chronic topical application in rats. PPD can also induce systemic immunologic effects in several organs and tissues or induce local inflammation [40].

We designed an animal study to evaluate the impact of a minimal PPD dose (60 μg/kg/day intraperitoneally) on bladder function and pathological changes. Our data demonstrated that PPD generated oxidative stress, caused subsequent tissue damage, and induced bladder ischemia and inflammation. These data indicated that PPD could induce local inflammation and ischemia in addition to a systemic immunological response. The urine ROS counts were not significantly increased after PPD exposure, which could be attributed to endogenous detoxification and defense mechanisms or less biologically toxic metabolites in the urine. The use of novel Ni-SOD mimics, WCT003 and WCT006, efficiently attenuated ROS generation, inflammation, apoptosis, autophagy, and pyroptosis in the PPD-treated bladders and improved bladder dysfunction. Synthetic Ni-SOD mimics could improve PPD-induced ROS related bladder injuries.

Prevalence of lower urinary tract symptoms (LUTS) and overactive bladder in male and female humans increases with advancing age and impacts the quality of life [41, 42]. A primary cause of chronic bladder/pelvic ischemia is bladder outflow obstruction (male) or atherosclerosis (male/female) [43]. Chronic bladder/pelvic ischemia resulted in oxidative stress that upregulated tissue-damage marker compounds, increased denervation of the bladder [43], and reduced the muscarinic receptor function in the urinary bladder [44]. Urothelial sensory signaling was also altered, including direct activation of C-fiber afferent neurons, urothelium hyperpermeability to inflammatory mediators, TRPM8 upregulation, and COX pathway excitation [45–47]. H_2_O_2_ acts as an endothelium-dependent vasoconstrictor in rat arteries that activates smooth muscle Ca^2+^ entry through L-type and non-L-type channels as well as intracellular signaling pathways, including the production of NADPH oxidase-derived superoxide [48]. Excess PPD-induced ROS production may cause vasoconstriction in the urinary bladder that impairs bladder nerves, urothelial cells, and receptors in smooth muscles, leading to inflammation and bladder dysfunction.

We demonstrated that a minimal dose of chronic intraperitoneal PPD successfully induced bladder ischemia and oxidative stress that mimicked the etiology of human LUTS. Our data indicated that PPD-induced oxidative stress led to detrusor hyperactivity. Chronic PPD administration was associated with the increased infiltration of neutrophils and mast cells and the expression of PARP, LC3 II, and caspase-1 markers in the hyperactive bladder. The synthetic Ni-SOD mimics, WCT003 and WCT006, efficiently reduced PPD-induced neutrophil infiltration, mast cells, ROS levels, and expression of PARP, LC3 II, and caspase-1 markers. Ni-SOD mimics partially ameliorated bladder hyperactivity. This was the first study to demonstrate the relationship between PPD and bladder dysfunction in an animal model with the histopathological changes that followed PPD exposure. Our results warrant further investigation of the clinical association between PPD and LUTS. The utility of the novel Ni-SOD mimics WCT003 and WCT006 should be investigated for the treatment of ROS-induced diseases.

In conclusion, we established a model for bladder hyperactivity induced by intraperitoneal PPD exposure in animals. We provided direct evidence that PPD-induced bladder hyperactivity resulted from ROS generation and bladder ischemia. We observed bladder inflammation and enhanced apoptosis, autophagy, and pyroptosis in the damaged bladders. The synthetic Ni-SOD mimics, WCT003 and WCT006, mitigated bladder inflammation and programmed cell death to improve PPD-induced bladder hyperactivity.

## MATERIALS AND METHODS

### Experimental animals

Twenty-four adult female Wistar rats weighing 220–250g were purchased from BioLASCO Taiwan Co. Ltd. (Taipei, Taiwan) and were housed at the Experimental Animal Center, National Taiwan Normal University, with a consistent light-dark cycle (12:12-hour) and temperature (36.5–37.0°C). Food and water were provided ad libitum. The rats were randomly divided into four groups by type of intraperitoneal injection as follows: 1) control, n=6: 4 weeks of 1 mL/day normal saline; 2) PPD, n=6: 4 weeks of PPD (60 μg/kg/day, n=6); 3) PPD+WCT003, n=6: 4 weeks of PPD (60 μg/kg/day) and 2 subsequent weeks of 1.5 mg/kg/day WCT003; 4. PPD+WCT006, n=6: 4 weeks of PPD (60 μg/kg/day) and 2 subsequent weeks of 1.5 mg/kg/day WCT006. The United States Environmental Protection Agency (EPA) calculated a provisional Reference Dose (RfD) of 0.19 mg/kg/day PPD based on whole-body effects in rats [49]. The highest cumulative PPD penetration obtained was 4.47 g/cm^2^, which yielded a 0.052 mg/kg systemic exposure dose (close to 60 μg/kg) according to SCCNFP/0129/99. We used a chronic minimal dose of 60 μg/kg/day intraperitoneally for 4 weeks compared to a previous experimental study that used a single dose [15]. The rats were placed in R-2100 metabolic cages (Lab Products, Rockville, Maryland) the day before surgical procedures to evaluate the fecal and urinary amount and voiding frequency. The rats were given free access to food and water during the metabolic cage studies.

All the surgical and experimental procedures were approved by the Institutional Animal Care and Use Committee of the National Taiwan Normal University and were in accordance with the guidelines of the National Science Council of Republic of China (NSC 1997). Animal care and experimental protocols were in accordance with the guidelines of the National Science Council of the Republic of China (1997).

### Preparation of Ni-SOD mimics

**Figure 7:**
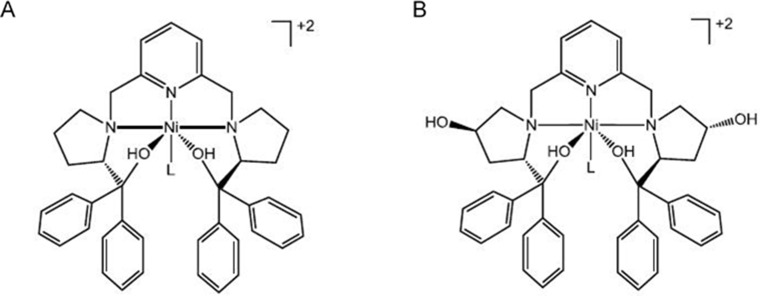
ChemDraw representations of Ni-SOD mimics **(A)** WCT003; **(B)** WCT006.

WCT003 and WCT006 were prepared by reacting [2,6-bis(((*S*)-2-(diphenylhydroxymethyl)-1-pyrrolidinyl)methyl)pyridine] or [2,6- bis(((*S*)-2-(diphenylhydroxymethyl)-4-hydroxy-1-pyrrolidinyl)methyl)pyridine], respectively, with [Ni(CH_3_CN)_6_](ClO_4_)_2_. The detailed preparation of WCT003 and WCT006 was described in U.S. Patent No. 8,642,763. WCT006 had a hydroxyl substituent on the pyrrolidine ring to increase the water solubility. WCT003 did not contain this hydroxyl substituent (Figure 7).

### Hemodynamic and cystometric parameters

The surgical procedures were described previously [50]. Briefly, PE-50 catheters were placed in the left femoral artery to measure arterial blood pressure (ABP) and in the left femoral vein for administration of drugs under anesthesia (subcutaneous urethane injection, 1.2 g/kg body weight). ABP was recorded by an ADI system (Power-Lab/16S, ADI Instruments, Pty., Ltd., Castle Hill, Australia) with a transducer (Gould-Statham, Quincy, USA). Body temperature was maintained at 36.5–37°C by an infrared light and monitored with a rectal thermometer.

We introduced a transcystometric model to evaluate the micturition alteration in the bladder in response to PPD stimulation. The method has been well-established in our laboratory [50]. Briefly, the bladder was exposed by a midline incision of the abdomen. A PE-50 bladder catheter was connected via T-tube to an infusion pump (0.04 mL/min rate) with a pressure transducer (Gould-Statham, Quincy, USA), and inserted through the apex of the bladder dome. The intravesical pressure (IVP) was continuously recorded by an ADI system (Power-Lab/16S). The following parameters of bladder responsiveness were measured: intercontraction interval (ICI, the time lag between two micturition cycles identified with active contractions (>15 mmHg)); baseline bladder pressure (BP); micturition time (MT); maximal voiding pressure (MVP); contractile amplitude (A=MVP-BP); and threshold pressure (PTH) for a micturition (Figure 1A). The change in IVP, ABP, and various parameters can be determined before and after PPD administration.

### Bladder microcirculation determination

A full-field laser perfusion imager (MoorFLPI, Moor Instruments Ltd., Devon, UK) was applied to monitor the microcirculatory blood-flow intensity continuously [51]. The imager used laser-speckle contrast imaging, which exploited the random speckle pattern that was generated when tissue was illuminated by laser light. The random speckle pattern changed when blood cells moved within the region of interest (ROI). When there was a high level of movement (fast flow), the changing pattern became more blurred, and the contrast in that region was reduced accordingly. The contrast image was processed to produce a 16-color-coded image that correlated with blood flow in the heart: blue was defined as low flow and red as high flow. The microcirculatory blood-flow intensity of each ROI was recorded as the flux in perfusion unit, which was related to the product of average speed and concentration of the moving red blood cells in the bladder sample volume. The negative control value was set at 0 perfusion units (blue color), and the positive control value was 1000 perfusion units (red color). The perfusion units were analyzed in real time by the MoorFLPI software version 3.0.

### *In vitro* ROS activity

On the day of treatment, the ROS activity in the blood and urine *in vitro* was measured by a luminol and lucigenin chemiluminescence detection method as described previously [50]. Briefly, 0.2 mL of whole blood or urine samples were mixed with 0.5 mL of 0.1 mmol/L lucigenin or 0.2 mmol/L luminal and analyzed with a Chemiluminescence Analyzing System (CLD-110, Tohoku Electronic Inc. Co., Sendai, Japan). Each assay was performed in triplicate and the chemiluminescence count emitted from the above reaction mixture was recorded per 10 sec for 300 sec. The recorded total signals corresponded to the H_2_O_2_ and HOCl count in the luminol method or the superoxide anion (O_2_¯) count in the lucigenin method and were obtained by measuring the total chemiluminescence from the area under the curve of chemiluminescence count in 300 sec.

### *In vivo* bladder surface O_2_¯ production

The ROS response on the bladder surface was measured *in vivo* by an isovolumetric model using intravenous infusion of the superoxide anion probe, 2-methyl-6-(4-methoxyphenyl)-3,7-dihydroimidazo-[1,2-a]-pyrazin-3-one-hydrochloride (MCLA) (0.2 mg/mL/hr, TCI-Ace, Tokyo Kasei Kogyo Co. Ltd, Tokyo, Japan), and recorded with a Chemiluminescence Analyzing System as described previously [52]. Production of O_2_¯ on the bladder surface was induced by intravenous infusion ofMCLA and recorded throughout the experiment. The anesthetized animal was housed in a dark box with a shielded plate to exclude photon emission from sources other than the exposed bladder. The bladder was unshielded and positioned under a reflector to reflect photons from the exposed bladder surface onto the detector. The MCLA-enhanced chemiluminescent signal from the bladder surface was recorded continuously by the chemiluminescence analyzer. The total O_2_¯ chemiluminescence value was measured by area under the curve from the bladder. The chemiluminescence of 0.2 mL saline in 1 mL of MCLA (0.2 mg/mL) served as the negative control and 0.2 mL xanthine (0.75 mg/kg body weight)/xanthine oxidase (24.8 mU/kg body weight) in 1 mL of MCLA (0.2 mg/mL) was the positive control [52]. TheMCLA-enhanced chemiluminescence was continuously recorded every 10 s. The real-time chemiluminescence signal from the O_2_¯ level on the bladder surface was detected.

### In situ detection of oxidative stress, inflammation, autophagy, apoptosis, and pyroptosis

We used a 3-nitrotyrosine (3-NT) assay to localize oxidative stress production, a toluidine blue stain for mast cell-mediated inflammation, an LC3 II stain for autophagy, terminal deoxynucleotidyl transferase-mediated nick-end labeling (TUNEL) for apoptosis, and caspase 1-staining to indicate pyroptosis in the paraffin-embedded bladder sections. Sections (5 μm) of bladders obtained after paraffin microtome (RM 2125 RTS, LEICA, Germany) were stained with hematoxylin and eosin for evaluation of the extent of leukocyte accumulation [52]. We incubated sections with toluidine blue solution (Polysciences, 1 g/100 mL of 70% ethanol stock) diluted 1:10 with 1 g/mL aqueous NaCl for 2 min followed by 3 rinses with deionized water. The selective mast cell stain demonstrated a blue color under microscopic observation. The bladder sections were prepared by incubation with a polyclonal antibody (Alpha Diagnostic International; San Antonio, TX, USA) diluted at 1:50 for the 3-NT evaluation. The value of brown deposits/total section area in the 3-NT assay was counted by Adobe Photoshop 7.0.1 image software analysis.

Deparaffinized sections (5 μm) were incubated with rabbit anti-LC3 II (diluted 1:500 in PBS, Cell Signaling Technology) and anti-caspase 1 (diluted 1:500 in PBS, Epitomics, Abcam, Cambridge, England), incubated overnight at 4°C, and washed with PBS three times (5 min each). Secondary antibodies (Super Sensitive TM Non-Biotin polymer HRP IHC) were used for detection (BioGenex, San Ramon, CA, USA). The signal was visualized by incubation with liquid diaminobenzidine tetrahydrochloride. TUNEL staining was used to measure DNA fragmentation in deparaffinized, fixed sections according to the manufacturer's protocol (FragEL DNA Fragmentation kit, Calbiochem), and the resulting sections were visualized by fluorescence microscopy [53]. For quantification, TUNEL-positive nuclei were counted in 5 randomly selected high-power (400EL fields, and an average was determined for each section.

### Apoptosis, autophagy, and pyroptosis protein expression

The expression levels of apoptosis-related proteins, poly (ADP-ribose) polymerase (PARP), autophagy-related protein LC3-II, and pyroptosis-related protein caspase 1 in bladder tissues were analyzed by Western blotting. The bladder samples were homogenized with a pre-chilled mortar and pestle in extraction buffer (10 mM Tris-HCl (pH 7.6), 140 mM NaCl, 1 mM PMSF, 1% NP-40, 0.5% deoxycholate, 2% β-mercaptoethanol, 10 μg/mL pepstatin A, and 10 μg/mL aprotinin). The homogenate was centrifuged at 12000 ×g for 12 min at 4°C, the supernatant was collected, and the protein concentrations were determined by a BioRad Protein Assay (BioRad Laboratories, Hercules, CA, USA). Antibodies raised against LC3-II (Epitomics, Abcam, Cambridge, England), caspase 1 (Epitomics, Abcam, Cambridge, England), PARP (Cell Signaling Technology, Inc.) and β-actin (Sigma, Saint Louis, MI) were used. SDS-PAGE was performed on 12.5% separation gels in the absence of urea and stained with Coomassie brilliant blue. Each lane contained 30 μg of total protein and was transferred to nitrocellulose filters. The immunoreactive bands were detected by incubation with the appropriate antibody described previously, followed by secondary antibody-alkaline phosphatase, and finally with NBT and 5-bromo-4chloro-3-indolyl phosphate in a toluidine salt (Roche Diagnostic GmbH, Mannheim, Germany) stock solution for 30 min at room temperature. The density of the band with the appropriate molecular mass was determined semi-quantitatively by densitometry using an image analyzing system (Alpha Innotech, San Leandro, CA, USA).

### Statistical analysis

SigmaPlot 10.0 (Systat Software, Inc., Chicago, IL, USA) software was used for graphing and statistical analysis. All values were expressed as the mean ± standard error. All parameters were compared within the control and PPD groups by using a Student's paired t-test. Two-way analysis of variance was used to establish differences among groups. Intergroup comparisons were made by Duncan's multiple-range test. A *P* < 0.05 indicated a significant difference.

## Abbreviations

3-NT: 3-nitrotyrosine
A: contractile amplitude
ABP: arterial blood pressure
BP: baseline bladder pressure
CL: chemiluminescence
EPA: Environmental Protection Agency
ICI: inter-contraction interval
IVP: intravesical pressure
IARC: International Agency for Research on Cancer
LC3 II: microtubule-associated protein 1A/1B-light chain 3-phosphatidylethanolamine conjugate
LUTS: lower urinary tract symptoms
MCLA: 2-methyl-6-(4-methoxyphenyl)-3,7-dihydroimidazo-[1,2-a]-pyrazin-3-one-hydrochloride
MT: micturition time
MVP: maximal voiding pressure
Ni-SOD: nickel-containing superoxide dismutases
PPD: para-phenylenediamine
PTH: threshold pressure
PARP: poly ADP-ribose polymerase
RfD: provisional Reference Dose
ROS: reactive oxygen species
SDS-PAGE: sodium dodecyl sulfate-polyacrylamide gel electrophoresis
SODs: superoxide dismutases
TUNEL: terminal deoxynucleotidyl transferase dUTP nick end labeling
